# Increasing access to therapy from primary care : The Finnish First-Line Therapies - initiative and stepped-care model

**DOI:** 10.1192/j.eurpsy.2026.10917

**Published:** 2026-07-31

**Authors:** S. Saarni, K. Mikkonen, J. Ekelund, S. Saarni

**Affiliations:** 1 MET, Tampere University; 2 Department of Psychiatry, Pirkanmaa Well-being services county, Tampere; 3 Department of Psychiatry, HUS, Helsinki; 4 Psychiatry, Päijät-Häme Wellbeing Services County, Lahti, Finland

## Abstract

**Introduction:**

Mental disorders pose a major global health burden, yet a persistent treatment gap leaves most people in need without adequate, timely and evidence-based treatments, especially psychotherapies. Despite two decades of WHO-led initiatives to scale up and invest in mental health services, progress remains limited. Most countries have not met mental health service targets due to chronic underinvestment, and have failed to scale up evidence-based psychosocial treatments to match population-level needs.

**Objectives:**

To describe Finland’s First-Line Therapies (FLT) initiative, a nationwide system-level initiative launched in 2020 to close the treatment gap through strengthening and digitally augmenting primary care mental health services.

**Methods:**

FLT was implemented as a change-management initiative within Finland’s sweeping 2023 health and social systems reform, emphasizing process redesign and digitalization over new treatments or increase of resources. Key strategies included a localized stepped-care model, digital tools for assessment and treatment, and a national e-learning platform for workforce competence development.

**Results:**

FLT was rapidly scaled up nationwide. Key components include a digital triage tool (Finnish Therapy Navigator, FTN), over 50 online self-help and guided self-help programs, a nationwide internet-based CBT service, and a national e-learning platform to train thousands of clinicians annually in evidence-based therapies.

The FTN is used by over 2% of the population annually, standardizing initial assessments to under 30 minutes and saving clinician time, with over 90% patient and 95% provider acceptance. The stepped-care model ensures low-intensity interventions are used first if appropriate, with higher-intensity therapy provided as needed. The nationwide iCBT service enables patients to start therapy within one week at no cost to them. A new e-learning platform has trained ca 16 000 thousand professionals in several brief evidence-based psychotherapies, greatly expanding workforce competence. Notably, these improvements in access and efficiency were achieved with minimal new spending.

**Conclusions:**

The FLT joins a growing list of models aiming to improve early access to evidence-based psychosocial treatments, spearheaded by the UK IAPT / NHS Talking therapies model. The Finnish experience shows that digitally augmented system reform can in some cases also narrow the mental health treatment gap without major new increases in staff. By focusing on scalable interventions and embedding changes into existing services, FLT addresses key barriers such as implementation failure, limited scalability, and underinvestment, and offers a WHO-aligned framework for reform that can be of intrest to other countries (including low-resource settings) in expanding access to evidence-based treatments within existing systems.

**Disclosure of Interest:**

None Declared

